# Assessment of Compliance by Mental Health Professionals on the General Adult Inpatient Wards in Mersey Care NHS Foundation Trust With DVLA Guidelines on Driving Restrictions for Individuals With a Mental Health Condition

**DOI:** 10.1192/bjo.2025.10603

**Published:** 2025-06-20

**Authors:** Kareem Salem, Declan Hyland

**Affiliations:** Mersey Care NHS Foundation Trust, Liverpool, United Kingdom

## Abstract

**Aims:** Individuals with a severe and enduring mental illness may pose a significant risk to themselves and others when they drive, particularly if in a state of relapse. The DVLA provides guidance for driving for individuals with mental disorders. It provides guidance for medical professionals caring for such patients to determine during their assessments the potential for safe driving and restrictions, if any. Patients admitted to a psychiatric ward who drive should be informed of the DVLA guidance and any restrictions on driving prior to discharge and such discussions should be documented in the inpatient’s medical record. This audit aimed to assess compliance of mental health professionals on the general adult inpatient wards with DVLA guidelines regarding patients about driving restrictions and whether such discussions are documented in the inpatient’s medical record.

**Methods:** A total of 58 patient discharges from three of the general adult inpatient wards in the Trust constituted the sample to be audited. An audit tool was designed to assess whether there was documentation of the following for each patient: mental disorder diagnosis, driving status, type of vehicle driven, patient informed that their mental disorder may affect their ability to drive safely, and advice on driving restrictions as stipulated by the DVLA. The patient’s electronic patient record was used to obtain all data retrospectively.

**Results:** All patients had their mental disorder diagnosis documented. Only 5 of the 58 patients had their driving status documented. None of the 58 patients had type of vehicle driven documented. None of the 58 patients were informed that their mental disorder may affect their ability to drive safely. None of the 58 patients were provided with advice on DVLA driving restrictions at the point of discharge from hospital.

**Conclusion:** This audit highlighted consideration of driving status and need for advice on driving restrictions at the time of discharge from the general adult inpatient ward as currently being an area of poor clinical practice despite the safety implications to both the patient, those they get in a car with and other road users. A recommendation was made for the Trust’s admission clerking to include a section on driving status and type of vehicle driven. A second recommendation was for doctors working on the general adult inpatient wards to be aware of DVLA guidance for individuals with mental disorder and the importance of discussing driving status during weekly ward reviews, especially at the time of discharge.

